# Synthesis and Antimicrobial Activity of 2-[2-(2,6-dichloro phenyl)amino]benzyl-3-(5-substituted phenyl-4,5-dihydro-1H-pyrazol-3-yl-amino)-6,8-dibromoquinazolin-4(3H)ones

**DOI:** 10.4103/0975-1483.63165

**Published:** 2010

**Authors:** NB Patel, JC Patel, GG Barat

**Affiliations:** *Department of Chemistry, Veer Narmad South Gujarat University, Surat 395007, Gujarat, India*

**Keywords:** Antimicrobial activity, chalcone, pyrazoline, quinazolin-4(3H)one

## Abstract

A series of 2-[2-(2,6-dichlorophenyl)amino]benzyl-3-(5-substituted phenyl-4,5-dihydro-1H-pyrazol-3-yl-amino)-6,8-dibromoquinazolin-4(3H) ones 6a-m have been synthesized by the reaction of 2-[2-(2,6-dichlorophenyl)amino]benzyl-3-substituted phenylacrylamido-6,8-dibromoquinazolin-4(3H) ones 5a-m with hydrazine hydrate in the presence of glacial acetic acid. The chalcones 5a-m were prepared by the condensation of 2-[2-(2,6-dichlorophenyl)amino]benzyl-3-acetamido-6,8-dibromoquinazolin-4(3H)one 4 with different substituted aromatic aldehyde. The benzoxazinone 2 was synthesized from 2-[2-(2,6-dichlorophenyl)amino]phenyl acetyl chloride 1 on treatment with 3,5-dibromoanthranilic acid in pyridine, which on reaction with hydrazine hydrate and then on acetylation reaction yielded 4. The structures of these compounds have been elucidated by elemental analyses, IR, and NMR spectral data. The title compounds pyrazolyl-quinazolin-4(3H)ones 6a-m were evaluated for their antibacterial and antifungal activities *in vitro*.

## INTRODUCTION

The recent literature reveals that the quinazolinone moiety associated with various aromatic as well heterocyclic compounds possess wide range of pharmacological properties such as antibacterial,[1] antifungal,[1] analgesic,[2] anti-inflammatory,[3] anthelminthic,[4] anticonvulsant,[5] anti HIV,[6] antitubercular,[7] CNS depressant,[8] cytotoxicity,[9] diuretic,[10] and hypolipidemic[11] activities. Pyrazoline systems are known to be biologically active and are important constituents of many pharmacological products. These compounds are known for their antibacterial,[12] antifungal,[13] antimycobacterial,[14] analgesic,[15] anti-inflammatory,[16] anticancer,[17] antiamoebic,[18] molluscicidal,[19] hypotensive,[20] antinociceptive,[21] antidepressant,[22] anticonvulsant,[23] and antiviral[24] activities. The aim of the present work was to attach pyrazoline molecule to quinazolin-4(3H)one in order to find new biologically active pharmacophore. Thus, synthesis of pyrazolyl-quinazolin-4(3H)ones 6a-m has been achieved. The potency[25] of compounds 6a-m is compared with standard drugs to study the strength of compounds 6a-m, with a hope to get a better antimicrobial agent.

## MATERIALS AND METHODS

The melting points were determined in open capillary tubes and are uncorrected. The infrared (IR) spectra of the synthesized compounds were recorded using KBr pellet on Perkin Elmer 1300 FTIR spectrometer and frequencies are recorded in wave number (cm^-1^). Nuclear magnetic resonance (^1^H-NMR and ^13^C-NMR) spectra were recorded on Bruker Avance II 400 NMR spectrometer using deutero chloroform (CDCl_3_) as a solvent. The chemical shifts are reported in δ (part per million) downfield from tetramethylsilane (TMS) as an internal standard. The purity of all the compounds was checked by TLC on Merck silica gel 60 F254 using toluene:ethylacetate (8:2) as mobile phase, and spots were visualized under UV radiation. 2-[(2,6-Dichlorophenyl)amino]phenyl acetyl chloride 1 was synthesized by the literature procedure.[26]

Procedure for the preparation of 2-[2-(2,6-Dichlorophenyl)amino]benzyl-6,8-dibromo-3,1-benzoxazin-4(H)one (2)

A mixture of 2-[(2,6-dichlorophenyl)amino]phenyl acetyl chloride 1 (0.01 mol) and 3,5-dibromo anthranilic acid (0.01 mol) in 20 ml pyridine was stirred at 0-5 °C for 1 h, further stirred for 1 h at room temperature. After completion of reaction, a pasty mass obtained and was washed thoroughly with sodium bicarbonate (5 % w/v) to remove unreacted acid. A solid separated was filtered, dried, and recrystallized from methanol.

Yield: 68%. m.p. 171-173 °C. IR (KBr) (cm^-1^): 3447 (NH str), 2927, 2854 (CH_2_str), 1748 (C=O str), 1614 (C=N str), 1150 (C-O str), 743 (C-Cl str), 565 (C-Br str). ^1^H-NMR (CDCl_3_, 400 MHz), δ (ppm): 3.55 (s, 2H, CH_2_), 6.37-8.15 (m, 9H, Ar-H), 9.13 (bs, 1H, NH). Anal. found: C, 45.36; H, 2.15; N, 5.07 %; Calcd. for C_21_H_12_Br_2_Cl_2_N_2_O_2_, C, 45.44; H, 2.18; N, 5.05 %.

Procedure for the preparation of 3-Amino-2-[2-(2,6-Dichlorophenyl)amino]benzyl-6,8-dibromoquinazolin-4(3H)one (3)

A mixture of 2-[2-(2,6-dichlorophenyl)amino]benzyl-6,8-dibromo-3,1-benzoxazine-4(H)one 2 (0.01 mol) and hydrazine hydrate (0.01 mol) in 25 ml absolute ethanol was refluxed on water bath for 6-8 h. After completion of the reaction, it was slowly poured onto crushed ice with continuous stirring. The solid thus obtained, was filtered, and washed several times with cold water. The crude product was dried and recrystallized from ethanol.

Yield: 64%. m.p. 146-148 °C. IR (KBr) (cm^-1^): 3510-3393 (NH and NH_2_str), 2932, 2857 (CH_2_str), 1721 (C=O str), 1612 (C=N str), 748 (C-Cl str), 570 (C-Br str). ^1^H-NMR (CDCl_3_, 400 MHz), δ (ppm): 3.58 (s, 2H, CH_2_), 5.75 (bs, 2H, NH_2_), 6.38-8.10 (m, 9H, Ar-H), 9.15 (bs, 1H, NH). Anal. found: C, 44.25; H, 2.53; N, 9.81 %; Calcd. for C_21_H_14_Br_2_Cl_2_N_4_O, C, 44.32; H, 2.48; N, 9.85 %.

Procedure for the preparation of 2-[2-(2,6-Dichlorophenyl)amino]benzyl-3-acetamido-6,8-dibromoquinazolin-4(3H)one (4)

To the solution of 3-amino-2-[2-(2,6-dichlorophenyl)amino]benzyl-6,8-dibromoquinazolin-4(3*H*)one **3** (0.01 mol) in 50 ml dry benzene, acetyl chloride (0.01 mol) was added drop by drop at 0-5 °C over the period of 1 h with continuous shaking. After completion of the addition, the reaction mixture was kept overnight. The excess of solvent was distilled off under reduced pressure and then poured onto ice and shaken well. The solid thus obtained was filtered and recrystallized from methanol.

Yield: 69 %. m.p. 193-195 °C. IR (KBr) (cm^-1^): 3447 (NH str), 2935, 2859 (CH_2_str), 1727 (C=O str), 1645(C=O str of amide), 1615 (C=N str), 753 (C-Cl str), 573 (C-Br str). ^1^H-NMR (CDCl_3_, 400 MHz), δ (ppm): 2.23 (s, 3H, CH_3_), 3.53 (s, 2H, CH_2_), 6.37-8.12 (m, 9H, Ar-H), 9.14 (bs, 1H, NH), 10.32 (bs, 1H, NH). Anal. found: C, 45.28; H, 2.61; N, 9.15 %; Calcd. for C_23_H_16_Br_2_Cl_2_N_4_O_2_, C, 45.20; H, 2.64; N, 9.17 %.

General procedure for the preparation of 2-[2-(2,6-Dichlorophenyl)amino]benzyl-3-(substituted phenylacrylamido)-6,8-dibromoquinazolin-4(3H)ones (5a-m)

To the solution of 2-[2-(2,6-dichlorophenyl)amino]benzyl-3-acetamido-6,8-dibromoquinazolin-4(3*H*)one **4** (0.01 mol) in 50 ml absolute ethanol, benzaldehyde (0.01 mol) in 2 % NaOH was added and refluxed for 10-12 h. After completed the reaction, it was concentrated, cooled, and poured onto ice. The solid thus obtained was filtered, washed with water, and recrystallized from methanol. The remaining compounds **5b-m** were synthesized by using the same procedure.

2-[2-(2,6-Dichlorophenyl)amino]benzyl-3-(phenylacrylamido)-6,8-dibromoquinazolin-4(3H)ones (5a)

Yield: 67 %. m.p. 146-148 °C. IR (KBr) (cm^-1^): 3441 (NH str), 2921, 2852 (CH_2_str), 1719 (C=O str), 1613 (C=N str), 1576 (CH=CH str), 749 (C-Cl str), 578 (C-Br str). ^1^H-NMR (CDCl_3_, 400 MHz), δ (ppm): 3.56 (s, 2H, CH_2_), 6.39-8.16 (m, 14H, Ar-H), 6.80 (d, 1H, =CHCO, *J* = 16 Hz), 7.62 (d, 1H, =CH-Ar, *J* = 16 Hz), 8.84 (bs, 1H, CONH), 9.18 (bs, 1H, NH). ^13^C-NMR (CDCl_3_, 100 MHz), δ (ppm): 30.57 (CH_2_), 112.37-148.53 (26C, CH=CH and Ar-C), 162.06 (C=O), 168.25 (C=N), 173.12 (CONH). Anal. found: C, 51.46; H, 2.92; N, 8.04 %; Calcd. for C_30_H_20_Br_2_Cl_2_N_4_O_2_, C, 51.53; H, 2.88; N, 8.01%.

2-[2-(2,6-Dichlorophenyl)amino]benzyl-3-(2-hydroxyphenylacrylamido)-6,8-dibromoquinazolin-4(3H)ones (5b)

Yield: 72%. m.p. 156-158 °C. IR (KBr) (cm^-1^): 3547 (OH str), 3438 (NH str), 2926, 2854 (CH_2_str), 1722 (C=O str), 1617 (C=N str), 1566 (CH=CH str), 757 (C-Cl str), 573 (C-Br str). ^1^H-NMR (CDCl_3_, 400 MHz), δ (ppm): 3.54 (s, 2H, CH_2_), 6.37-8.13 (m, 13H, Ar-H), 6.78 (d, 1H, =CHCO, *J* = 16.2 Hz), 7.59 (d, 1H, =CH-Ar, *J* = 16.2 Hz), 8.82 (bs, 1H, CONH), 9.16 (bs, 1H, NH), 10.36 (bs, 1H, OH). ^13^C-NMR (CDCl_3_, 100 MHz), δ (ppm): 30.63 (CH_2_), 112.23-155.67 (26C, CH=CH and Ar-C), 161.82 (C=O), 168.40 (C=N), 173.22 (CONH). Anal. found: C, 50.35; H, 2.86; N, 7.79 %; Calcd. for C_30_H_20_Br_2_Cl_2_N_4_O_3_, C, 50.38; H, 2.82; N, 7.83 %.

2-[2-(2,6-Dichlorophenyl)amino]benzyl-3-(3-hydroxyphenylacrylamido)-6,8-dibromoquinazolin-4(3H)ones (5c)

Yield: 66%. m.p. 171-173 °C. IR (KBr) (cm^-1^): 3542 (OH str), 3450 (NH str), 2930, 2855 (CH_2_str), 1726 (C=O str), 1612 (C=N str), 1575 (CH=CH str), 752 (C-Cl str), 580 (C-Br str). ^1^H-NMR (CDCl_3_, 400 MHz), δ (ppm): 3.51 (s, 2H, CH_2_), 5.57 (bs, 1H, OH), 6.37-8.14 (m, 13H, Ar-H), 6.80 (d, 1H, =CHCO, *J* = 16.2 Hz), 7.63 (d, 1H, =CH-Ar, *J* = 16.2 Hz), 8.86 (bs, 1H, CONH), 9.15 (bs, 1H, NH). ^13^C-NMR (CDCl_3_, 100 MHz), δ (ppm): 30.60 (CH_2_), 112.18-159.29 (26C, CH=CH and Ar-C), 162.12 (C=O), 168.17 (C=N), 172.98 (CONH). Anal. found: C, 50.31; H, 2.77; N, 7.88 %; Calcd. for C_30_H_20_Br_2_Cl_2_N_4_O_3_, C, 50.38; H, 2.82; N, 7.83 %.

2-[2-(2,6-Dichlorophenyl)amino]benzyl-3-(4-hydroxyphenylacrylamido)-6,8-dibromoquinazolin-4(3H)ones (5d)

Yield: 73 %. m.p. 184-186 °C. IR (KBr) (cm^-1^): 3548 (OH str), 3446 (NH str), 2934, 2853 (CH_2_str), 1720 (C=O str), 1609 (C=N str), 1572 (CH=CH str), 748 (C-Cl str), 581 (C-Br str). ^1^H-NMR (CDCl_3_, 400 MHz), δ (ppm): 3.58 (s, 2H, CH_2_), 5.59 (bs, 1H, OH), 6.38-8.12 (m, 13H, Ar-H), 6.81 (d, 1H, =CHCO, *J* = 16.4 Hz), 7.62 (d, 1H, =CH-Ar, *J* = 16.4 Hz), 8.80 (bs, 1H, CONH), 9.15 (bs, 1H, NH). ^13^C-NMR (CDCl_3_, 100 MHz), δ (ppm): 30.45 (CH_2_), 112.23-157.32 (26C, CH=CH and Ar-C), 161.93 (C=O), 168.03 (C=N), 173.34 (CONH). Anal. found: C, 50.42; H, 2.83; N, 7.76%; Calcd. for C_30_H_20_Br_2_Cl_2_N_4_O_3_, C, 50.38; H, 2.82; N, 7.83%.

2-[2-(2,6-Dichlorophenyl)amino]benzyl-3-(2-chlorophenylacrylamido)-6,8-dibromoquinazolin-4(3H)ones (5e)

Yield: 72 %. m.p. 145-147 °C. IR (KBr) (cm^-1^): 3444 (NH str), 2922, 2846 (CH_2_str), 1723 (C=O str), 1614 (C=N str), 1578 (CH=CH str), 745 (C-Cl str), 583 (C-Br str). ^1^H-NMR (CDCl_3_, 400 MHz), δ (ppm): 3.55 (s, 2H, CH_2_), 6.37-8.13 (m, 13H, Ar-H), 6.78 (d, 1H, =CHCO, *J* = 16.4 Hz), 7.61 (d, 1H, =CH-Ar, *J* = 16.4 Hz), 8.81 (bs, 1H, CONH), 9.13 (bs, 1H, NH). ^13^C-NMR (CDCl_3_, 100 MHz), δ (ppm): 30.26 (CH_2_), 111.95-148.49 (26C, CH=CH and Ar-C), 162.02 (C=O), 168.18 (C=N), 173.27 (CONH). Anal. found: C, 49.20; H, 2.67; N, 7.61 %; Calcd. for C_30_H_19_Br_2_Cl_3_N_4_O_2_, C, 49.11; H, 2.61; N, 7.64 %.

2-[2-(2,6-Dichlorophenyl)amino]benzyl-3-(3-chlorophenylacrylamido)-6,8-dibromoquinazolin-4(3H)ones (5f)

Yield: 65 %. m.p. 163-165 °C. IR (KBr) (cm^-1^): 3451 (NH str), 2926, 2849 (CH_2_str), 1718 (C=O str), 1611 (C=N str), 1580 (CH=CH str), 755 (C-Cl str), 580 (C-Br str). ^1^H-NMR (CDCl_3_, 400 MHz), δ (ppm): 3.52 (s, 2H, CH_2_), 6.38-8.15 (m, 13H, Ar-H), 6.82 (d, 1H, =CHCO, *J* = 16.2 Hz), 7.60 (d, 1H, =CH-Ar, *J* = 16.2 Hz), 8.79 (bs, 1H, CONH), 9.16 (bs, 1H, NH). ^13^C-NMR (CDCl_3_, 100 MHz), δ (ppm): 30.42 (CH_2_), 112.08-148.36 (26C, CH=CH and Ar-C), 161.87 (C=O), 167.92 (C=N), 173.14 (CONH). Anal. found: C, 49.06; H, 2.57; N, 7.68 %; Calcd. for C_30_H_19_Br_2_Cl_3_N_4_O_2_, C, 49.11; H, 2.61; N, 7.64 %.

2-[2-(2,6-Dichlorophenyl)amino]benzyl-3-(4-chlorophenylacrylamido)-6,8-dibromoquinazolin-4(3H)ones (5g)

Yield: 69 %. m.p. 176-178 °C. IR (KBr) (cm^-1^): 3447 (NH str), 2930, 2855 (CH_2_str), 1716 (C=O str), 1610 (C=N str), 1576 (CH=CH str), 744 (C-Cl str), 571 (C-Br str). ^1^H-NMR (CDCl_3_, 400 MHz), δ (ppm): 3.58 (s, 2H, CH_2_), 6.37-8.13 (m, 13H, Ar-H), 6.84 (d, 1H, =CHCO, *J* = 16 Hz), 7.60 (d, 1H, =CH-Ar, *J* = 16 Hz), 8.82 (bs, 1H, CONH), 9.17 (bs, 1H, NH). ^13^C-NMR (CDCl_3_, 100 MHz), δ (ppm): 30.55 (CH_2_), 112.12-148.59 (26C, CH=CH and Ar-C), 162.14 (C=O), 167.88 (C=N), 173.03 (CONH). Anal. found: C, 49.17; H, 2.65; N, 7.58 %; Calcd. for C_30_H_19_Br_2_Cl_3_N_4_O_2_, C, 49.11; H, 2.61; N, 7.64 %.

2-[2-(2,6-Dichlorophenyl)amino]benzyl-3-(2-nitrophenylacrylamido)-6,8-dibromoquinazolin-4(3H)ones (5h)

Yield: 70%. m.p. 202-204°C. IR (KBr) (cm^-1^): 3454 (NH str), 2932, 2856 (CH_2_str), 1723 (C=O str), 1615 (C=N str), 1582 (CH=CH str), 1544, 1361 (NO_2_str), 745 (C-Cl str), 577 (C-Br str). ^1^H-NMR (CDCl_3_, 400 MHz), δ (ppm): 3.54 (s, 2H, CH_2_), 6.38-8.16 (m, 13H, Ar-H), 6.83 (d, 1H, =CHCO, *J* = 16.2 Hz), 7.62 (d, 1H, =CH-Ar, *J* = 16.2 Hz), 8.81 (bs, 1H, CONH), 9.16 (bs, 1H, NH). ^13^C-NMR (CDCl_3_, 100 MHz), δ (ppm): 30.38 (CH_2_), 112.06-150.29 (26C, CH=CH and Ar-C), 162.25 (C=O), 168.04 (C=N), 172.91 (CONH). Anal. found: C, 48.36; H, 2.52; N, 9.46%; Calcd. for C_30_H_19_Br_2_Cl_2_N_5_O_4_, C, 48.42; H, 2.57; N, 9.41%.

2-[2-(2,6-Dichlorophenyl)amino]benzyl-3-(3-nitrophenylacrylamido)-6,8-dibromoquinazolin-4(3H)ones (5i)

Yield: 68%. m.p. 227-229°C. IR (KBr) (cm^-1^): 3453 (NH str), 2930, 2856 (CH_2_str), 1722 (C=O str), 1606 (C=N str), 1576 (CH=CH str), 1538, 1356 (NO_2_str), 741 (C-Cl str), 573 (C-Br str). ^1^H-NMR (CDCl_3_, 400 MHz), δ (ppm): 3.52 (s, 2H, CH_2_), 6.37-8.40 (m, 13H, Ar-H), 6.82 (d, 1H, =CHCO, *J* = 16 Hz), 7.64 (d, 1H, =CH-Ar, *J* = 16 Hz), 8.84 (bs, 1H, CONH), 9.15 (bs, 1H, NH). ^13^C-NMR (CDCl_3_, 100 MHz), δ (ppm): 30.35 (CH_2_), 112.23-150.44 (26C, CH=CH and Ar-C), 161.98 (C=O), 168.28 (C=N), 172.84 (CONH). Anal. found: C, 48.34; H, 2.60; N, 9.43%; Calcd. for C_30_H_19_Br_2_Cl_2_N_5_O_4_, C, 48.42; H, 2.57; N, 9.41%.

2-[2-(2,6-Dichlorophenyl)amino]benzyl-3-(4-nitrophenylacrylamido)-6,8-dibromoquinazolin-4(3H)ones (5j)

Yield: 63%. m.p. 238-239°C. IR (KBr) (cm^-1^): 3446 (NH str), 2932, 2857 (CH_2_str), 1726 (C=O str), 1608 (C=N str), 1585 (CH=CH str), 1543, 1362 (NO_2_str), 756 (C-Cl str), 582 (C-Br str). ^1^H-NMR (CDCl_3_, 400 MHz), δ (ppm): 3.58 (s, 2H, CH_2_), 6.38-8.16 (m, 13H, Ar-H), 6.80 (d, 1H, =CHCO, *J* = 16.2 Hz), 7.61 (d, 1H, =CH-Ar, *J* = 16.2 Hz), 8.82 (bs, 1H, CONH), 9.16 (bs, 1H, NH). ^13^C-NMR (CDCl_3_, 100 MHz), δ (ppm): 30.57 (CH_2_), 112.18-148.24 (26C, CH=CH and Ar-C), 162.21 (C=O), 168.07 (C=N), 173.22 (CONH). Anal. found: C, 48.49; H, 2.51; N, 9.37%; Calcd. for C_30_H_19_Br_2_Cl_2_N_5_O_4_, C, 48.42; H, 2.57; N, 9.41%.

2-[2-(2,6-Dichlorophenyl)amino]benzyl-3-(4-dimethylaminophenylacrylamido)-6,8-dibromo quinazolin-4(3H)ones (5k)

Yield: 67%. m.p. 161-163 °C. IR (KBr) (cm^-1^): 3448 (NH str), 2933, 2858 (CH_2_str), 1718 (C=O str), 1612 (C=N str), 1579 (CH=CH str), 752 (C-Cl str), 578 (C-Br str). ^1^H-NMR (CDCl_3_, 400 MHz), δ (ppm): 2.84 (s, 6H, N(CH_3_)_2_), 3.55 (s, 2H, CH_2_), 6.37-8.15 (m, 13H, Ar-H), 6.78 (d, 1H, =CHCO, *J* = 16.4 Hz), 7.59 (d, 1H, =CH-Ar, *J* = 16.4 Hz), 8.78 (bs, 1H, CONH), 9.13 (bs, 1H, NH). ^13^C-NMR (CDCl_3_, 100 MHz), δ (ppm): 30.27 (CH_2_), 46.64 (N-(CH_3_)_2_), 111.92-150.13 (26C, CH=CH and Ar-C), 162.14 (C=O), 168.12 (C=N), 173.06 (CONH). Anal. found: C, 51.67; H, 3.32; N, 9.47%; Calcd. for C_32_H_25_Br_2_Cl_2_N_5_O_2_, C, 51.78; H, 3.39; N, 9.43%.

2-[2-(2,6-Dichlorophenyl)amino]benzyl-3-(2-methoxyphenylacrylamido)-6,8-dibromo quinazolin-4(3H)ones (5l)

Yield: 65 %. m.p. 157-159 °C. IR (KBr) (cm^-1^): 3453 (NH str), 2928, 2850 (CH_2_str), 1723 (C=O str), 1615 (C=N str), 1580 (CH=CH str), 1245, 1103 (C-O-C str), 751, (C-Cl str), 570 (C-Br str). ^1^H-NMR (CDCl_3_, 400 MHz), δ (ppm): 3.52 (s, 2H, CH_2_), 3.65 (s, 3H, OCH_3_), 6.38-8.15 (m, 13H, Ar-H), 6.82 (d, 1H, =CHCO, *J* = 16 Hz), 7.63 (d, 1H, =CH-Ar, *J* = 16 Hz), 8.80 (bs, 1H, CONH), 9.18 (bs, 1H, NH). ^13^C-NMR (CDCl_3_, 100 MHz), δ (ppm): 30.64 (CH_2_), 61.15 (OCH_3_), 112.23-156.34 (26C, CH=CH and Ar-C), 162.26 (C=O), 167.79 (C=N), 173.35 (CONH). Anal. found: C, 50.92; H, 3.10; N, 7.62 %; Calcd. for C_31_H_22_Br_2_Cl_2_N_4_O_3_, C, 51.06; H, 3.04; N, 7.68 %.

2-[2-(2,6-Dichlorophenyl)amino]benzyl-3-(4-methoxyphenylacrylamido)-6,8-dibromo quinazolin-4(3H)ones (5m)

Yield: 68%. m.p. 173-175°C. IR (KBr) (cm^-1^): 3442 (NH str), 2923, 2849 (CH_2_str), 1710 (C=O str), 1609 (C=N str), 1581 (CH=CH str), 1243, 1108 (C-O-C str), 745 (C-Cl str), 569 (C-Br str). ^1^H-NMR (CDCl_3_, 400 MHz), δ (ppm): 3.51 (s, 2H, CH_2_), 3.66 (s, 3H, OCH_3_), 6.37-8.16 (m, 13H, Ar-H), 6.80 (d, 1H, =CHCO, *J* = 16.2 Hz), 7.58 (d, 1H, =CH-Ar, *J* = 16.2 Hz), 8.78 (bs, 1H, CONH), 9.16 (bs, 1H, NH). ^13^C-NMR (CDCl_3_, 100 MHz), δ (ppm): 30.57 (CH_2_), 59.38 (OCH_3_), 111.97-158.29 (26C, CH=CH and Ar-C), 161.97 (C=O), 167.86 (C=N), 173.16 (CONH). Anal. found: C, 51.14; H, 3.01; N, 7.67%; Calcd. for C_31_H_22_Br_2_Cl_2_N_4_O_3_, C, 51.06; H, 3.04; N, 7.68%.

General procedure for the preparation of 2-[2-(2,6-Dichlorophenyl)amino]benzyl-3-(5-substituted phenyl-4,5-dihydro-1H-pyrazol-3-yl-amino)-6,8-dibromoquinazolin-4(3H)ones (6a-m)

A mixture of 2-[2-(2,6-dichlorophenyl)amino]benzyl-3-(phenylacrylamido)-6,8-dibromo quinazolin-4(3*H*)one **5a** (0.01 mol) and hydrazine hydrate (0.01 mol) in 30 ml absolute methanol was added few drops of glacial acetic acid and refluxed for 8-10 h. After completion of the reaction, excess of solvent was distilled off; the separated solid was filtered, washed with water, and recrystallized from methanol. Similarly other pyrazolyl derivatives **6b-m** were synthesized.

2-[2-(2,6-Dichlorophenyl)amino]benzyl-3-(5-phenyl-4,5-dihydro-1H-pyrazol-3-yl-amino)-6,8-dibromoquinazolin-4(3H)ones (6a)

Yield: 58 %. m.p. 133-135 °C. IR (KBr) (cm^-1^): 3443 (NH str), 2924, 2853 (CH_2_str), 1727 (C=O str), 1612 (C=N str), 747 (C-Cl str), 575 (C-Br str). ^1^H-NMR (CDCl_3_, 400 MHz), δ (ppm): 3.04 (dd, 1H, Ha, *J*_a,b_ = 17.4 Hz, *J*_ax_ = 5.5 Hz), 3.47 (dd, 1H, Hb, *J*_ba_ = 17.4 Hz, *J*_bx_ = 12 Hz), 3.54 (s, 2H, CH_2_), 5.49 (dd, 1H, Hx, *J*_xb_ = 12 Hz, *J*_xa_= 5.5 Hz), 6.38-8.15 (m, 15H, NH and Ar-H), 8.35 (bs, 1H, NH), 9.17 (bs, 1H, NH). ^13^C-NMR (CDCl_3_, 100 MHz), δ (ppm): 30.46 (CH_2_), 36.42 (CH_2_of pyrazole), 55.57 (CH of pyrazole), 112.43-148.57 (24C, Ar-C), 161.17 (C=N of pyrazole), 162.15 (C=O), 168.34 (C=N). Anal. found: C, 50.46; H, 3.08; N, 11.72 %; Calcd. for C_30_H_22_Br_2_Cl_2_N_6_O, C, 50.52; H, 3.11; N, 11.78 %.

2-[2-(2,6-Dichlorophenyl)amino]benzyl-3-[5-(2-hydroxyphenyl)-4,5-dihydro-1H-pyrazol-3-yl-amino]-6,8-dibromoquinazolin-4(3H)ones (6b)

Yield: 62 %. m.p. 149-151 °C. IR (KBr) (cm^-1^): 3543 (OH str), 3440 (NH str), 2927, 2853 (CH_2_str), 1732 (C=O str), 1607 (C=N str), 755 (C-Cl str), 578 (C-Br str). ^1^H-NMR (CDCl_3_, 400 MHz), δ (ppm): 3.02 (dd, 1H, Ha, *J*_a,b_= 17.4 Hz, J_ax_= 5.5 Hz), 3.45 (dd, 1H, Hb, *J*_ba_= 17.4 Hz, *J*_bx_= 11.9 Hz), 3.56 (s, 2H, CH_2_), 5.48 (dd, 1H, Hx, *J*_xb_= 11.9 Hz, *J*_xa_= 5.5 Hz), 6.37-8.14 (m, 14H, NH and Ar-H), 8.42 (bs, 1H, NH), 9.15 (bs, 1H, NH), 10.35 (bs, 1H, OH). ^13^C-NMR (CDCl_3_, 100 MHz), δ (ppm): 30.60 (CH_2_), 35.41 (CH_2_of pyrazole), 55.65 (CH of pyrazole), 112.19-155.63 (24C, Ar-C), 161.21 (C=N of pyrazole), 161.94 (C=O), 168.29 (C=N). Anal. found: C, 49.38; H, 3.03; N, 11.54 %; Calcd. for C_30_H_22_Br_2_Cl_2_N_6_O_2_, C, 49.41; H, 3.04; N, 11.52 %.

2-[2-(2,6-Dichlorophenyl)amino]benzyl-3-[5-(3-hydroxyphenyl)-4,5-dihydro-1H-pyrazol-3-yl-amino]-6,8-dibromoquinazolin-4(3H)ones (6c)

Yield: 67 %. m.p. 162-163 °C. IR (KBr) (cm^-1^): 3540 (OH str), 3453 (NH str), 2928, 2854 (CH_2_str), 1730 (C=O str), 1609 (C=N str), 750 (C-Cl str), 578 (C-Br str). ^1^H-NMR (CDCl_3_, 400 MHz), δ (ppm): 3.05 (dd, 1H, Ha, *J*_a,b_= 17.6 Hz, *J*_ax_= 5.4 Hz), 3.46 (dd, 1H, Hb, *J*_ba_= 17.6 Hz, *J*_bx_= 12 Hz), 3.53 (s, 2H, CH_2_), 5.48 (dd, 1H, Hx, *J*_xb_= 12 Hz, *J*_xa_= 5.4 Hz), 5.59 (bs, 1H, OH), 6.37-8.15 (m, 14H, NH and Ar-H), 8.37 (bs, 1H, NH), 9.14 (bs, 1H, NH). ^13^C-NMR (CDCl_3_, 100 MHz), δ (ppm): 30.48 (CH_2_), 36.53 (CH_2_of pyrazole), 54.98 (CH of pyrazole), 112.23-159.33 (24C, Ar-C), 161.13 (C=N of pyrazole), 162.26 (C=O), 168.09 (C=N). Anal. found: C, 49.33; H, 3.08; N, 11.55 %; Calcd. for C_30_H_22_Br_2_Cl_2_N_6_O_2_, C, 49.41; H, 3.04; N, 11.52 %.

2-[2-(2,6-Dichlorophenyl)amino]benzyl-3-[5-(4-hydroxyphenyl)-4,5-dihydro-1H-pyrazol-3-yl-amino]-6,8-dibromoquinazolin-4(3H)ones (6d)

Yield: 63 %. m.p. 177-179 °C. IR (KBr) (cm^-1^): 3550 (OH str), 3448 (NH str), 2931, 2852 (CH_2_str), 1725 (C=O str), 1608 (C=N str), 743 (C-Cl str), 583 (C-Br str). ^1^H-NMR (CDCl_3_, 400 MHz), δ (ppm): 3.07 (dd, 1H, Ha, *J*_a,b_= 17.4 Hz, *J*_ax_= 5.4 Hz), 3.49 (dd, 1H, Hb, *J*_ba_= 17.4 Hz, *J*_bx_= 11.8 Hz), 3.56 (s, 2H, CH_2_), 5.46 (dd, 1H, Hx, *J*_xb_= 11.8 Hz, *J*_xa_= 5.4 Hz), 5.58 (bs, 1H, OH), 6.37-8.12 (m, 14H, NH and Ar-H), 8.40 (bs, 1H, NH), 9.17 (bs, 1H, NH). ^13^C-NMR (CDCl_3_, 100 MHz), δ (ppm): 30.39 (CH_2_), 36.51 (CH_2_of pyrazole), 55.62 (CH of pyrazole), 112.18-157.38 (24C, Ar-C), 161.34 (C=N of pyrazole), 162.04 (C=O), 167.97 (C=N). Anal. found: C, 49.46; H, 3.05; N, 11.48 %; Calcd. for C_30_H_22_Br_2_Cl_2_N_6_O_2_, C, 49.41; H, 3.04; N, 11.52 %.

2-[2-(2,6-Dichlorophenyl)amino]benzyl-3-[5-(2-chlorophenyl)-4,5-dihydro-1H-pyrazol-3-yl-amino]-6,8-dibromoquinazolin-4(3H)ones (6e)

Yield: 59 %. m.p. 143-145 °C. IR (KBr) (cm^-1^): 3442 (NH str), 2924, 2847 (CH_2_str), 1720 (C=O str), 1611 (C=N str), 741 (C-Cl str), 585 (C-Br str). ^1^H-NMR (CDCl_3_, 400 MHz), δ (ppm): 3.02 (dd, 1H, Ha, *J*_a,b_= 17.2 Hz, *J*_ax_= 5.5 Hz), 3.46 (dd, 1H, Hb, *J*_ba_= 17.2 Hz, *J*_bx_= 12 Hz), 3.54 (s, 2H, CH_2_), 5.48 (dd, 1H, Hx, *J*_xb_= 12 Hz, *J*_xa_= 5.5 Hz), 6.38-8.14 (m, 14H, NH and Ar-H), 8.38 (bs, 1H, NH), 9.15 (bs, 1H, NH). ^13^C-NMR (CDCl_3_, 100 MHz), δ (ppm): 30.32 (CH_2_), 36.46 (CH_2_of pyrazole), 55.56 (CH of pyrazole), 112.03-148.57 (24C, Ar-C), 161.25 (C=N of pyrazole), 162.13 (C=O), 168.10 (C=N). Anal. found: C, 48.12; H, 2.81; N, 11.25 %; Calcd. for C_30_H_21_Br_2_Cl_3_N_6_O, C, 48.19; H, 2.83; N, 11.24 %.

2-[2-(2,6-Dichlorophenyl)amino]benzyl-3-[5-(3-chlorophenyl)-4,5-dihydro-1H-pyrazol-3-yl-amino]-6,8-dibromoquinazolin-4(3H)ones (6f)

Yield: 67 %. m.p. 151-153 °C. IR (KBr) (cm^-1^): 3447 (NH str), 2926, 2850 (CH_2_str), 1723 (C=O str), 1613 (C=N str), 749 (C-Cl str), 579 (C-Br str). ^1^H-NMR (CDCl_3_, 400 MHz), δ (ppm): 3.04 (dd, 1H, Ha, *J*_a,b_= 17.4 Hz, *J*_ax_= 5.5 Hz), 3.47 (dd, 1H, Hb, *J*_ba_= 17.4 Hz, *J*_bx_= 11.8 Hz), 3.52 (s, 2H, CH_2_), 5.46 (dd, 1H, Hx, *J*_xb_= 11.8 Hz, *J*_xa_= 5.5 Hz), 6.38-8.15 (m, 14H, NH and Ar-H), 8.40 (bs, 1H, NH), 9.13 (bs, 1H, NH). ^13^C-NMR (CDCl_3_, 100 MHz), δ (ppm): 30.24 (CH_2_), 36.56 (CH_2_of pyrazole), 55.63 (CH of pyrazole), 112.17-148.66 (24C, Ar-C), 161.20 (C=N of pyrazole), 162.18 (C=O), 168.21 (C=N). Anal. found: C, 48.26; H, 2.79; N, 11.22 %; Calcd. for C_30_H_21_Br_2_Cl_3_N_6_O, C, 48.19; H, 2.83; N, 11.24 %.

2-[2-(2,6-Dichlorophenyl)amino]benzyl-3-[5-(4-chlorophenyl)-4,5-dihydro-1H-pyrazol-3-yl-amino]-6,8-dibromoquinazolin-4(3H)ones (6g)

Yield: 63 %. m.p. 166-168 °C. IR (KBr) (cm^-1^): 3440 (NH str), 2921, 2848 (CH_2_str), 1719 (C=O str), 1608 (C=N str), 743 (C-Cl str), 575 (C-Br str). ^1^H-NMR (CDCl_3_, 400 MHz), δ (ppm): 3.06 (dd, 1H, Ha, *J*_a,b_= 17.5 Hz, *J*_ax_= 5.4 Hz), 3.49 (dd, 1H, Hb, *J*_ba_= 17.5 Hz, *J*_bx_= 11.9 Hz), 3.54 (s, 2H, CH_2_), 5.48 (dd, 1H, Hx, *J*_xb_= 11.9 Hz, *J*_xa_= 5.4 Hz), 6.37-8.16 (m, 14H, NH and Ar-H), 8.37 (bs, 1H, NH), 9.14 (bs, 1H, NH). ^13^C-NMR (CDCl_3_, 100 MHz), δ (ppm): 30.38 (CH_2_), 36.42 (CH_2_of pyrazole), 55.53 (CH of pyrazole), 111.98-148.57 (24C, Ar-C), 161.23 (C=N of pyrazole), 162.29 (C=O), 168.36 (C=N). Anal. found: C, 48.08; H, 2.80; N, 11.28 %; Calcd. for C_30_H_21_Br_2_Cl_3_N_6_O, C, 48.19; H, 2.83; N, 11.24 %.

2-[2-(2,6-Dichlorophenyl)amino]benzyl-3-[5-(2-nitrophenyl)-4,5-dihydro-1H-pyrazol-3-yl-amino]-6,8-dibromoquinazolin-4(3H)ones (6h)

Yield: 69 %. m.p. 169-171 °C. IR (KBr) (cm^-1^): 3450 (NH str), 2928, 2852 (CH_2_str), 1725 (C=O str), 1612 (C=N str), 1548, 1363 (NO_2_str), 754 (C-Cl str), 572 (C-Br str). ^1^H-NMR (CDCl_3_, 400 MHz), δ (ppm): 3.04 (dd, 1H, Ha, *J*_a,b_= 17.6 Hz, *J*_ax_= 5.5 Hz), 3.47 (dd, 1H, Hb, *J*_ba_= 17.6 Hz, *J*_bx_= 12 Hz), 3.52 (s, 2H, CH_2_), 5.46 (dd, 1H, Hx, *J*_xb_= 12 Hz, *J*_xa_= 5.5 Hz), 6.39-8.16 (m, 14H, NH and Ar-H), 8.41 (bs, 1H, NH), 9.15 (bs, 1H, NH). ^13^C-NMR (CDCl_3_, 100 MHz), δ (ppm): 30.21 (CH_2_), 36.59 (CH_2_of pyrazole), 55.68 (CH of pyrazole), 112.10-150.38 (24C, Ar-C), 161.12 (C=N of pyrazole), 162.35 (C=O), 168.19 (C=N). Anal. found: C, 47.43; H, 2.75; N, 12.89 %; Calcd. for C_30_H_21_Br_2_Cl_2_N_7_O_3_, C, 47.52; H, 2.79; N, 12.93 %.

2-[2-(2,6-Dichlorophenyl)amino]benzyl-3-[5-(3-nitrophenyl)-4,5-dihydro-1H-pyrazol-3-yl-amino]-6,8-dibromoquinazolin-4(3H)ones (6i)

Yield: 61 %. m.p. 187-189 °C. IR (KBr) (cm^-1^): 3455 (NH str), 2930, 2855 (CH_2_str), 1727 (C=O str), 1615 (C=N str), 1542, 1360 (NO_2_str), 750 (C-Cl str), 577 (C-Br str). ^1^H-NMR (CDCl_3_, 400 MHz), δ (ppm): 3.02 (dd, 1H, Ha, *J*_a,b_= 17.5 Hz, *J*_ax_= 5.4 Hz), 3.45 (dd, 1H, Hb, *J*_ba_= 17.5 Hz, *J*_bx_= 11.8 Hz), 3.53 (s, 2H, CH_2_), 5.47 (dd, 1H, Hx, *J*_xb_= 11.8 Hz, *J*_xa_= 5.4 Hz), 6.38-8.39 (m, 14H, NH and Ar-H), 8.42 (bs, 1H, NH), 9.17 (bs, 1H, NH). ^13^C-NMR (CDCl_3_, 100 MHz), δ (ppm): 30.27 (CH_2_), 36.52 (CH_2_of pyrazole), 55.65 (CH of pyrazole), 112.13-150.46 (24C, Ar-C), 161.05 (C=N of pyrazole), 162.23 (C=O), 168.14 (C=N). Anal. found: C, 47.45; H, 2.76; N, 12.92 %; Calcd. for C_30_H_21_Br_2_Cl_2_N_7_O_3_, C, 47.52; H, 2.79; N, 12.93 %.

2-[2-(2,6-Dichlorophenyl)amino]benzyl-3-[5-(4-nitrophenyl)-4,5-dihydro-1H-pyrazol-3-yl-amino]-6,8-dibromoquinazolin-4(3H)ones (6j)

Yield: 63 %. m.p. 198-199 °C. IR (KBr) (cm^-1^): 3441 (NH str), 2925, 2852 (CH_2_str), 1729 (C=O str), 1611 (C=N str), 1540, 1363 (NO_2_str), 754 (C-Cl str), 587 (C-Br str). ^1^H-NMR (CDCl_3_, 400 MHz), δ (ppm): 3.05 (dd, 1H, Ha, *J*_a,b_= 17.4 Hz, *J*_ax_= 5.4 Hz), 3.48 (dd, 1H, Hb, *J*_ba_= 17.4 Hz, *J*_bx_= 12 Hz), 3.57 (s, 2H, CH_2_), 5.50 (dd, 1H, Hx, *J*_xb_= 12 Hz, *J*_xa_= 5.4 Hz), 6.37-8.15 (m, 14H, NH and Ar-H), 8.40 (bs, 1H, NH), 9.15 (bs, 1H, NH). ^13^C-NMR (CDCl_3_, 100 MHz), δ (ppm): 30.53 (CH_2_), 36.47 (CH_2_of pyrazole), 55.70 (CH of pyrazole), 112.24-148.39 (24C, Ar-C), 161.17 (C=N of pyrazole), 162.34 (C=O), 168.18 (C=N). Anal. found: C, 47.40; H, 2.80; N, 12.85 %; Calcd. for C_30_H_21_Br_2_Cl_2_N_7_O_3_, C, 47.52; H, 2.79; N, 12.93 %.

2-[2-(2,6-Dichlorophenyl)amino]benzyl-3-[5-(4-dimethylaminophenyl)-4,5-dihydro-1H-pyrazol-3-yl-amino]-6,8-dibromoquinazolin-4(3H)ones (6k)

Yield: 60 %. m.p. 157-159 °C. IR (KBr) (cm^-1^): 3454 (NH str), 2935, 2857 (CH_2_str), 1721 (C=O str), 1616 (C=N str), 758 (C-Cl str), 568 (C-Br str). ^1^H-NMR (CDCl_3_, 400 MHz), δ (ppm): 3.06 (dd, 1H, Ha, *J*_a,b_= 17.6 Hz, *J*_ax_= 5.5 Hz), 3.48 (dd, 1H, Hb, *J*_ba_= 17.6 Hz, *J*_bx_= 11.9 Hz), 3.56 (s, 2H, CH_2_), 5.51 (dd, 1H, Hx, *J*_xb_= 11.9 Hz, *J*_xa_= 5.5 Hz), 6.38-8.16 (m, 14H, NH and Ar-H), 8.37 (bs, 1H, NH), 9.14 (bs, 1H, NH). ^13^C-NMR (CDCl_3_, 100 MHz), δ (ppm): 30.32 (CH_2_), 36.38 (CH_2_of pyrazole), 55.59 (CH of pyrazole), 112.15-150.27 (24C, Ar-C), 161.22 (C=N of pyrazole), 162.28 (C=O), 168.24 (C=N). Anal. found: C, 50.75; H, 3.59; N, 12.98 %; Calcd. for C_32_H_27_Br_2_Cl_2_N_7_O, C, 50.82; H, 3.60; N, 12.96 %.

2-[2-(2,6-Dichlorophenyl)amino]benzyl-3-[5-(2-methoxyphenyl)-4,5-dihydro-1H-pyrazol-3-yl-amino]-6,8-dibromoquinazolin-4(3H)ones (6l)

Yield: 63 %. m.p. 147-149 °C. IR (KBr) (cm^-1^): 3440 (NH str), 2920, 2846 (CH_2_str), 1715 (C=O str), 1606 (C=N str), 1248, 1107 (C-O-C str), 759 (C-Cl str), 575 (C-Br str). ^1^H-NMR (CDCl_3_, 400 MHz), δ (ppm): 3.02 (dd, 1H, Ha, *J*_a,b_= 17.4 Hz, *J*_ax_= 5.3 Hz), 3.45 (dd, 1H, Hb, *J*_ba_= 17.4 Hz, *J*_bx_= 11.7 Hz), 3.51 (s, 2H, CH_2_), 3.66 (s, 3H, OCH_3_), 5.48 (dd, 1H, Hx, *J*_xb_= 11.7 Hz, *J*_xa_= 5.3 Hz), 6.37-8.15 (m, 14H, NH and Ar-H), 8.38 (bs, 1H, NH), 9.17 (bs, 1H, NH). ^13^C-NMR (CDCl_3_, 100 MHz), δ (ppm): 30.51 (CH_2_), 36.46 (CH_2_of pyrazole), 55.47 (CH of pyrazole), 112.11-156.26 (24C, Ar-C), 161.12 (C=N of pyrazole), 162.21 (C=O), 167.94 (C=N). Anal. found: C, 50.02; H, 3.21; N, 11.36 %; Calcd. for C_31_H_24_Br_2_Cl_2_N_6_O_2_, C, 50.09; H, 3.25; N, 11.31 %.

2-[2-(2,6-Dichlorophenyl)amino]benzyl-3-[5-(4-methoxyphenyl)-4,5-dihydro-1H-pyrazol-3-yl-amino]-6,8-dibromoquinazolin-4(3H)ones (6m)

Yield: 69 %. m.p. 165-167°C. IR (KBr) (cm^-1^): 3437 (NH str), 2918, 2845 (CH_2_str), 1718 (C=O str), 1607 (C=N str), 1238, 1105 (C-O-C str), 737 (C-Cl str), 562 (C-Br str). ^1^H-NMR (CDCl_3_, 400 MHz), δ (ppm): 3.03 (dd, 1H, Ha, *J*_a,b_= 17.5 Hz, *J*_ax_= 5.5 Hz), 3.46 (dd, 1H, Hb, *J*_ba_= 17.5 Hz, *J*_bx_= 11.8 Hz), 3.52 (s, 2H, CH_2_), 3.64 (s, 3H, OCH_3_), 5.49 (dd, 1H, Hx, *J*_xb_= 11.8 Hz, *J*_xa_= 5.5 Hz), 6.36-8.16 (m, 14H, NH and Ar-H), 8.42 (bs, 1H, NH), 9.15 (bs, 1H, NH). ^13^C-NMR (CDCl_3_, 100 MHz), δ (ppm): 30.64 (CH_2_), 36.40 (CH_2_of pyrazole), 55.42 (CH of pyrazole), 111.85-158.32 (24C, Ar-C), 160.97 (C=N of pyrazole), 162.05 (C=O), 168.02 (C=N). Anal. found: C, 49.96; H, 3.20; N, 11.24 %; Calcd. for C_31_H_24_Br_2_Cl_2_N_6_O_2_, C, 50.09; H, 3.25; N, 11.31 %.

### Antimicrobial activity

The *in vitro* antimicrobial activities of compounds 6a-m were carried out by the cup-plate method.[27] Antibacterial activity was screened against two gram-positive bacteria *S. aureus* (ATCC 12228) and B. subtilis (ATCC 11778), and two gram-negative bacteria E. coli (ATCC 8739) and Certium (ATCC 27957), by measuring the zone of inhibition on agar plates at two different concentrations 100 μg/ml and 50 μg/ml. While antifungal activity was tested by measuring the zone of inhibition on agar plates with two fungal species C. albicans (ATCC 10231) and A. niger (ATCC 16404) at two different concentrations 20 μg/ml and 10 μg/ml. Penicillin-G was used as a standard antibacterial agent, whereas fluconazole was used as a standard antifungal agent.

## RESULTS AND DISCUSSION

**Scheme 1:**
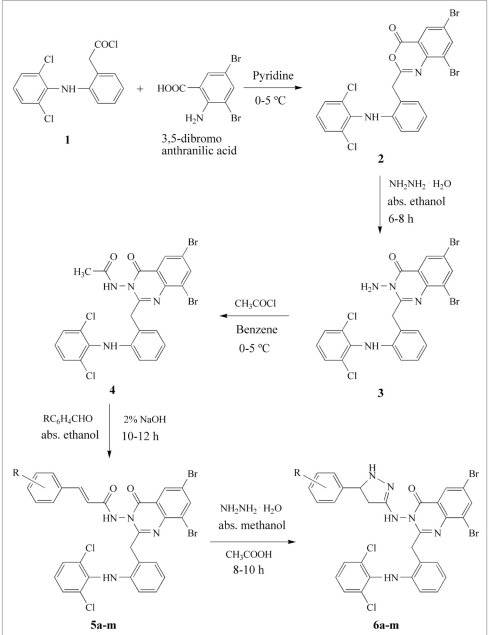
Synthetic route of compounds 6a-m

The title compounds 2-[2-(2,6-dichlorophenyl)amino]benzyl-3-(5-substituted phenyl-4,5-dihydro -1*H*-pyrazol-3-yl-amino)-6,8-dibromoquinazolin-4(3*H*)ones 6a-m were synthesized according to described process in Scheme 1. The structures of all the synthesized compounds were elucidated by the elemental analysis as well as IR and NMR spectral data. IR spectra showed strong C=O and C=N stretching of quinazolinone at around 1720 cm^-1^and 1610 cm^-1^. The ^1^H NMR spectra of compounds 6a-m indicated that the -CH_2_protons of the pyrazoline ring resonated as a pair of doublet of doublets (Ha and Hb) due to geminal and vicinal coupling. The CH proton appeared as a doublet of doublet (Hx) due to vicinal coupling with the two magnetically nonequivalent protons of methylene group at position 4 of pyrazoline ring. The Ha proton which is *cis* to Hx resonates upfield in the range δ 3.02-3.07 as a doublet of doublet while Hb, the other proton which is *trans* to Hx resonates downfield in the range δ 3.45-3.49 as a doublet of doublet. The Hx proton which is vicinal to two methylene protons (Ha and Hb) resonates as a doublet of doublet in the range δ 5.46-5.51. In ^13^C NMR spectra, signals at around δ 36.5, δ 55.5, and δ 161 confirms the presence of CH_2_, CH, and C=N of pyrazoline ring, respectively, whereas C=O and C=N signals of quinazolinone ring are appear at around δ 162 and δ 168, respectively.

The results of antibacterial activity are shown in Table 1. Compounds 6a (R = H) and 6h (R = 2-NO_2_) showed good activities against gram-positive bacteria (70.87% and 63.39% against *S. aureus* respectively; 73.97% and 69.56% against *B. subtilis*, respectively). The remaining compounds showed moderate activities (44.19-55.29%) against gram-positive bacteria as compared to penicillin-G. Compounds containing *para*-substituted hydroxyl, chloro and methoxy groups were found active than *ortho*- and *meta*-substituted compounds while *ortho* substituted nitro compound showed good activity than *meta*- and *para*-substituted nitro compounds against gram-positive bacteria. Compounds 6h (R = 2-NO_2_) and 6i (R = 3-NO_2_) exhibited good activities against gram-negative bacteria (65.04% and 74% against *E. coli*, respectively; 68.97% and 78.63% against *Certium*, respectively). The remaining compounds showed moderate activities (37.98-58.63%) against gram-negative bacteria as compared to penicillin-G. *Meta*-substituted hydroxyl and nitro compounds possessed good activity against gram-negative bacteria as compared to *ortho*- and *para*-substituted compounds. Whereas *ortho*-substituted chloro compound was found good as compared to meta and *para*, and methoxy group containing compounds share an equal activity against gram-negative bacteria *E. coli*. On the other hand, *ortho*-substituted methoxy compound was found good than *para*-substituted compound and *meta*-substituted chloro compound showed lower activity as compared to *ortho*- and *para*-substituted chloro compound against gram-negative bacteria *Certium*. Furthermore, compound 6h (R = 2-NO_2_) was active against all gram positive as well as gram-negative bacteria, while compound 6a (R = H) was active against both gram-positive bacteria and compound 6i (R = 3-NO_2_) was active against both gram-negative bacteria. Also, compound 6l (R = 2-OCH_3_) displayed good activity (61.15 %) against gram-negative bacteria *Certium*. In addition compounds containing dimethylamino group exhibited quite low activity than others against gram-positive as well as gram-negative bacteria.

**Table 1:** Antibacterial activity of compounds 6a-m

Compound	R	Zone of inhibition (mm)											
		*S. aureus* ATCC 12228			*B. subtilis* ATCC 11778			*E. coli* ATCC 8739			*Certium* ATCC 27957		
		C_H_	C_L_	Pot. %	C_H_	C_L_	Pot. %	C_H_	C_L_	Pot. %	C_H_	C_L_	Pot. %
6a	H	21	17	70.87	20	16	73.97	12	10	40.08	11	9	41.85
6b	2-OH	15	12	52.83	14	12	50.10	16	13	52.45	15	13	52.40
6c	3-OH	13	10	48.23	12	10	44.19	17	14	55.02	16	13	58.22
6d	4-OH	15	12	52.83	15	13	53.35	16	13	52.45	15	12	55.43
6e	2-Cl	13	10	48.23	12	10	44.19	14	12	44.63	13	11	46.83
6f	3-Cl	13	10	48.23	12	10	44.19	12	10	40.08	11	9	41.85
6g	4-Cl	14	11	50.47	13	11	47.05	13	11	42.30	13	11	46.83
6h	2-NO_2_	19	16	63.39	19	16	69.56	20	16	65.04	19	15	68.97
6i	3-NO_2_	15	12	52.83	14	12	50.10	23	19	74.00	22	18	78.63
6j	4-NO_2_	16	13	55.29	15	13	53.35	17	14	55.02	16	13	58.22
6k	4-N(CH_3_)_2_	13	10	48.23	12	10	44.19	11	9	37.98	10	8	39.56
6l	2-OCH_3_	14	11	50.47	13	11	47.05	18	15	57.71	17	14	61.15
6m	4-OCH_3_	15	12	52.83	14	12	50.10	18	15	57.71	17	15	58.63
Penicillin		30	25	100	27	21	100	31	25	100	28	23	100

C_H_ = zone of inhibition at 100 μg/ml

C_L_ = zone of inhibition at 50 μg/ml

Pot. = potency in %

The results of antifungal activity are shown in Table 2. Compounds 6d (R = 4-OH), 6e (R = 2-Cl), 6f (R = 3-Cl), 6g (R = 4-Cl), and 6h (R = 2-NO_2_) exhibited very good activities (61.84-84.70 %) against *C. albicans* while compounds 6e (R = 2-Cl), 6f (R = 3-Cl), and 6g (R = 4-Cl) possessed very good activities (63.41-78.57 %) against *A. niger* as compared to standard drug fluconazole. The remaining compounds showed moderate activities. Compounds 6e (R = 2-Cl) and 6g (R = 4-Cl) exhibited pronounced activities (84.70 % and 80.77 %) against *C. albicans* among the series. *Ortho*-substituted chloro and nitro compounds showed good activity as compared to *meta*- and *para*-substituted compounds against both *C. albicans* and *A. niger*. On the other hand, para-substituted hydroxyl compound was found good against *C. albicans* as compared to *ortho* and *meta*, while *meta*-substituted hydroxyl compound possessed higher activity against *A. niger* than *ortho*- and *para*-substituted compounds. Both ortho and *para*-substituted methoxy group containing compounds exhibited same activity against *C. albicans* and *A. niger*.

**Table 2:** Antifungal activity of compounds 6a-m

Compound	R	Zone of inhibition (mm)											
		*C. albicans* ATCC 10231			*A. niger* ATCC 16404		
		C_H_	C_L_	Pot. %	C_H_	C_L_	Pot. %
6a	H	15	12	58.63	15	13	51.52
6b	2-OH	10	8	41.28	9	7	35.88
6c	3-OH	7	0	49.07	6	0	41.30
6d	4-OH	9	7	68.84	9	7	35.88
6e	2-Cl	22	18	84.70	22	18	78.57
6f	3-Cl	19	16	72.59	18	15	63.41
6g	4-Cl	21	17	80.77	20	16	71.35
6h	2-NO2	16	13	61.84	15	13	51.52
6i	3-NO2	13	11	49.57	12	10	43.00
6j	4-NO2	15	13	56.01	14	12	48.51
6k	4-N(CH3)2	15	13	56.01	15	13	51.52
6l	2-OCH3	7	0	49.07	6	0	41.30
6m	4-OCH3	7	0	49.07	6	0	41.30
Fluconazole		26	21	100	28	22	100

C_H_ = zone of inhibition at 20 μg/ml

C_L_ = zone of inhibition at 10 μg/ml

Pot. = potency in %

## CONCLUSIONS

All the compounds showed satisfactory elemental as well as IR and NMR spectral results. Compounds bearing 2-nitro group showed promising activity against all bacterial species while chloro group-containing compounds were found active against both fungal species. All this findings give ideas to improve antimicrobial activity for further studies.
